# Economic burden and health-related quality of life in tenosynovial giant-cell tumour patients in Europe: an observational disease registry

**DOI:** 10.1186/s13023-021-01883-5

**Published:** 2021-07-02

**Authors:** J. Lopez-Bastida, I. Aranda-Reneo, B. Rodríguez-Sánchez, L. M. Peña-Longobardo, X. Ye, P. Laeis, E. M. Fronk, E. Palmerini, A. Leithner, M. A. J. Van de Sande

**Affiliations:** 1 Faculty of Health Sciences, University of Castilla-La Mancha, 45600 Talavera de la Reina, Toledo Spain; 2 Economic Analysis and Finance Department, Faculty of Social Sciences, University of Castilla-La Mancha, Avda. Real Fábrica de Seda S/N, 45600 Talavera de la Reina, Toledo Spain; 3 Faculty of Communication and Humanities, Faculty of Science and Technology, University Camilo José Cela, Madrid, Spain; 4 Economic Analysis and Finance Department, Faculty of Law and Social Sciences, University of Castilla-La Mancha, 45071 Toledo, Spain; 5 Daiichi Sankyo Inc., Basking Ridge, USA; 6 Daiichi Sankyo Europe, München, Germany; 7 IRCCS Istituto Ortopedico Rizzoli, Bologna, Italy; 8 Department of Orthopaedics and Trauma, Medical University of Graz, Graz, Austria; 9 Leiden University Medical Center, Leiden, The Netherlands

**Keywords:** Tenosynovial giant-cell tumour (TGCT), Cost-of-illness, Health-related quality of life, Economic burden, Productivity loss, Informal care, Europe

## Abstract

**Background:**

Tenosynovial Giant-Cell Tumour (TGCT) is a benign clonal neoplastic proliferation arising from the synovium, causing a variety of symptoms and often requiring repetitive surgery. This study aims to define the economic burden—from a societal perspective—associated with TGCT patients and their health-related quality of life (HRQOL) in six European countries.

**Methods:**

This article analyses data from a multinational, multicentre, prospective observational registry, the TGCT Observational Platform Project (TOPP), involving hospitals and tertiary sarcoma centres from six European countries (Austria, France, Germany, Italy, the Netherlands, and Spain). It includes information on TGCT patients’ health-related quality of life and healthcare and non-healthcare resources used at baseline (the 12-month period prior to the patients entering the registry) and after 12 months of follow-up.

**Results:**

146 TGCT patients enrolled for the study, of which 137 fulfilled the inclusion criteria. Their mean age was 44.5 years, and 62% were female. The annual average total costs associated with TGCT were €4866 at baseline and €5160 at the 12-month follow-up visit. The annual average healthcare costs associated with TGCT were €4620 at baseline, of which 67% and 18% corresponded to surgery and medical visits, respectively. At the 12-month follow-up, the mean healthcare costs amounted to €5094, with surgery representing 70% of total costs. Loss of productivity represented, on average, 5% of the total cost at baseline and 1.3% at follow-up. The most-affected HRQOL dimensions, measured with the EQ-5D-5L instrument, were pain or discomfort, mobility, and the performance of usual activities, both at baseline and at the follow-up visit. Regarding HRQOL, patients declared a mean index score of 0.75 at baseline and 0.76 at the 12-month follow-up.

**Conclusion:**

The results suggest that TGCT places a heavy burden on its sufferers, which increases after one year of follow-up, mainly due to the healthcare resources required—in particular, surgical procedures. As a result, this condition has a high economic impact on healthcare budgets, while the HRQOL of TGCT patients substantially deteriorates over time.

**Supplementary Information:**

The online version contains supplementary material available at 10.1186/s13023-021-01883-5.

## Introduction

The World Health Organisation’s classification of Tumours of Soft Tissue and Bone (2020) distinguishes two types of Tenosynovial Giant-Cell Tumour (TGCT): localised and diffuse lesions [1]. Microscopically, the two types show no clear differences. However, magnetic resonance imaging (MRI) discriminates between these two types [2]—in the diffuse type, there is a predilection for weight-bearing extremities, particularly the knee and the hip [3].

TGCTs are rare and usually affect young patients (adults between 30 and 50 years of age with a male/female ratio of about 1:1.5) [4]. However, although rare, TGCTs are probably under-reported and under-diagnosed, with an estimated overall annual incidence in the United States of 11 cases per million—1.8 cases per million for diffuse-type TGCT and 9.2 cases per million for localised TGCT [5]. The aggregate incidence rate in European countries is estimated at 5 cases per million [6]. A Danish study on localised and diffuse TGCT patients showed that their incidence rates per million person years were 30.3 and 8.4, respectively. Prevalence per 100,000 people was 44.3 for localised TGCT and 11.5 for diffuse TGCT [7]**.**

The current treatment of choice for TGCT-related symptoms is surgical excision, either by arthroscopic or open synovectomy [8]. Recurrence rates after surgical resection are high—up to 50% for localised and 80% for diffuse TGCT [9, 10].

Additionally, patients might experience a significant decline in their quality of life due to repetitive surgery, which may sometimes result in a partial loss of functioning of the affected joint and may also be associated with perioperative morbidity and secondary arthrosis. So far, no systemic treatment has been approved for this rare disease [11]**.** Consequently, TGCT causes pain, disability, and reduced work productivity, which explains its economic impact and its effects on quality of life, despite its low prevalence. For the United States, there is currently only one study on the economic impact of TGCT in terms of healthcare costs for the United States [12], while evidence on its consequences for health-related quality of life (HRQOL) is very limited [4, 13–15]. In the European context, information on the social costs and effects of TGCT on HRQOL is scarce or incomplete. This study aims to fill this gap, examining the societal, healthcare costs and HRQOL in patients with TGCT in six European countries. Thus, our aim was to obtain a solid estimate of the global burden of the disease from a societal perspective (including healthcare and non-healthcare costs and productivity losses). This information could be used to allocate research resources and to assess the actual relevance of the different intervention programmes targeting the disease.

## Methods

### Research design and subjects

A multinational, multicentre, prospective, non-interventional, observational disease registry, the TGCT Observational Platform Project (TOPP), was launched in November 2016, involving hospitals and tertiary sarcoma centres (recruitment sites) in six European countries (Austria, France, Germany, Italy, the Netherlands, and Spain). The study followed the Strengthening the Reporting of Observational Studies in Epidemiology (STROBE) guidelines [16]. Fieldwork was carried out between 2016 and 2019. Criteria for inclusion in the study were as follows: providing written informed consent for participation, above 18 years of age, patients with diffuse TGCT (histologically diagnosed), confirmed naïve or recurrent case. The study was approved by the Ethics Committees at each participating centre.

### Variables of interest

Data on TGCT patients (demographic information and current and historical clinical data) were collected at baseline from patients previously diagnosed with TGCT. This took place when the patient first attended the recruitment site and agreed to participate by signing the informed consent form. The outcomes reported by patients about TGCT symptoms and health-related quality of life were assessed at that time, together with the healthcare resources used in the past 12 months. When the patient returned for the follow-up visit after 12 months, any change in the data collected at baseline was recorded, together with patient-reported outcomes and health resource utilisation.

To estimate resource utilisation, the disease registry included information covering from the baseline period (12 months before the inclusion date) up to 12 months afterwards, at the time of the follow-up visit. Patients were also asked about reductions in their working time and work-related problems due to the disease. These data were used to estimate productivity losses. In addition, when care was provided by non-professional caregivers, they were asked about the informal care time. Information on HRQOL was collected from the disease registry through the generic EQ-5D tool [17].

### Costing method

We used the prevalence approach to estimate costs from a societal perspective. Prevalence-based cost-of-illness analysis has the advantage of including measurements of total annual healthcare expenditure—particularly relevant for chronic conditions such as TGCT, which require long-term treatment. A bottom-up costing approach was used to estimate total and average annual costs [18]. Data on resource utilisation were collected for each patient. When necessary, we used EUROSTAT’s Harmonised Index of Consumer Prices (HICP) to calculate inflated unit costs at €2019 values for each country. Since all participants were located in the euro area, unit costs were expressed in euros and exchange conversion was not necessary.

### Direct healthcare costs

Direct healthcare costs were calculated from healthcare services utilisation. Information on the number of hospital admissions, outpatient care (GPs, visits to specialists, physiotherapy sessions, and rehabilitation days), and surgery was also obtained from the patients through the disease registry. These resources were regarded as direct healthcare costs. Unit costs were obtained from different European databases recording healthcare costs and were subsequently multiplied by the number of units of each resource used. Both primary and secondary sources, including published papers, reports, and hospital accounting systems were used to collect the unit costs. A detailed list is provided in Table S1 (Additional file 1).

### Direct non-healthcare costs

For the purposes of this study, we only included informal care costs as direct non-healthcare costs. Informal care was defined as the help provided by non-professionals performing tasks that help maintain or enhance patients’ autonomy. Informal services were thus defined as the set of tasks performed, or the care provided by non-professional caregivers—often relatives, but sometimes friends or neighbours. Information on informal care was obtained from the disease registry—i.e., items regarding time spent helping the patient conduct basic activities [19]. The approach used to calculate the value of care hours was the good proxy method, which assesses time spent by the informal caregiver as an output. This method values the care provided by the informal caregiver by considering that if they did not provide these services, another person would have to do it [20]. In other words, this technique considers how much it would cost to substitute or replace the informal caregiver by hiring a professional. Thus, the value of informal care was calculated using a wage rate: the care hours that caregivers reported in the survey were identified; then, we calculated the value of these care hours considering different professional caregiver wage rates, depending on the selected country. Data on unit costs were provided by different sources in the six different countries (see Additional file 1: Table S1).

### Loss of productivity

Data about losses of productivity were also obtained from the registry, focusing on sick leave and early retirement due to TGCT. We used the human capital-based approach to estimate the productivity loss. Workers’ average earnings (gross wage) in the participating countries, as provided by EUROSTAT (see Additional file 1: Table S1), were used as a good proxy in the valuation of productivity losses [21].

### Patient outcomes

Patient outcomes were assessed using the EQ-5D-5L questionnaire [17], which has been validated for economic evaluation and health technology assessment in many countries in Europe [22]. This tool considers five dimensions of HRQOL: mobility, self-care, everyday activities, pain/discomfort, and anxiety/depression [23]. Through a descriptive, self-report system, an index or utility score can be estimated to measure overall health—in which 0 corresponds to death and 1 corresponds to perfect health, with negative values being possible. We used the mapping function developed by Van Hout et al. (2012) as reference-case analyses to estimate the utility scores or utility index that can be obtained using the EQ-5D tool [24]. The second part of the EQ-5D-5L consists of a zero-to-one-hundred Visual Analogue Scale (VAS), where 0 represents the worst and 100 represents the best imaginable state of health. Respondents placed a dot on the scale to reflect their overall perception of their health on the day they were included in the registry and another dot at the 12-month follow-up.

### Statistical methods

Descriptive analysis was carried out using mean and standard deviation (SD) in continuous variables and proportions for dichotomous or categorical variables. We used the two-sample t-test assuming unequal variances to compare the change on continuous variables from baseline to the endpoint (12-month visits). All analyses were carried out with Stata SE (v 14.2) [25]. Because TGCT is a rare disease, and recruitment of patients was completed within the schedule, no formal sample size consideration was performed.

## RESULTS

A total of 146 TGCT patients enrolled in the study. However, three participants withdrew their informed consent and six did not provide any information at the 12-month follow-up. Therefore, the final analysis consisted of 137 individuals diagnosed with TGCT. Their mean age was 45 years. The majority of them were female (62%), with secondary education (43%), and in employment (57%). Regarding TGCT severity, 54% participants had severe diffuse TGCT, 33% moderate diffuse TGCT, and 13% were not assessable.

In terms of HRQOL at the baseline time point, patients reported a mean 0.75 index score, or 68.34 points on the VAS. By country, France had the highest index score (0.85), followed by Spain (0.77), Austria (0.79), the Netherlands (0.76), Italy (0.75), and Germany (0.61) (Table 1). In terms of HRQOL at the 12-month follow-up, patients reported a mean 0.76 index score, or 71.27 points on the VAS, a slightly better score than in the baseline period (Table 1). By country, Spain had the highest score (0.86), followed by France (0.8), the Netherlands (0.77), Italy (0.76), Austria (0.75), and Germany (0.62) (Table 1). No statistically significant differences were observed between baseline and 12-month visit on VAS (mean difference = –2.93 points on VAS from baseline to 12-month visit; t = –1.15, p-value = 0.25) or utility index (mean difference = –0.01 on utility index from baseline to 12-month visit; t = –0.4, p-value = 0.69).

**Table 1:** Characteristics of study participants and health-related quality of life by country

	AUT (n = 9)	GER (n = 12)	SP (n = 13)	FRA (n = 4)	ITA (n = 38)	NLD (n = 61)	ALL (n = 137)
Age, mean (sd)	39.44 (19.25)	45 (13.44)	43.46 (13.93)	55 (22.2)	47.79 (13.92)	42.67 (13.63)	44.52 (14.44)
Gender, % female	67%	50%	62%	100%	61%	62%	62%
Highest level of education*, %							
Primary school	78%	8%	38%	0%	8%	8%	15%
Secondary school	11%	50%	23%	50%	26%	61%	43%
University	11%	42%	38%	50%	42%	31%	35%
Employment status**, % employed	44%	67%	62%	0%	63%	56%	57%
*TGCT Severity*							
Not assessable	44%	8%	38%	25%	3%	10%	13%
Moderate diffuse	44%	50%	23%	50%	47%	20%	33%
Severe diffuse	11%	42%	38%	25%	50%	70%	54%
*Health-related quality of life*							
EQ-5D index score at baseline, mean (sd)	0.79 (0.25)	0.61 (0.27)	0.77 (0.21)	0.85 (0.15)	0.75 (0.24)	0.76 (0.15)	0.75 (0.21)
EQ VAS Score at baseline, mean (sd)	69.44 (15.7)	61.25 (25.24)	64.85 (24.28)	78 (16.31)	68.13 (23.63)	69.83 (19.96)	68.34 (21.48)
EQ-5D index score at follow-up, mean (sd)	0.75 (0.36)	0.62 (0.34)	0.86 (0.09)	0.8 (0.22)	0.76 (0.26)	0.77 (0.18)	0.76 (0.24)
EQ VAS Score at follow-up, mean (sd)	74.38 (16.78)	58.64 (26.93)	81.58 (17.81)	76.25 (14.36)	72 (17.74)	70.25 (18.03)	71.27 (19.11)

AUT = Austria; GER = Germany; SP = Spain; FRA = France; ITA = Italy; NLD = The Netherlands

*Nine missing values

**Two missing values

Regarding the assessment of HRQOL dimensions at baseline with the EQ-5D-5L questionnaire, 37.7% of patients experienced at least moderate difficulty with mobility; 11.1% had at least moderate difficulty with self-care; 37% suffered at least moderate difficulty when performing usual activities; 48.8% had at least moderate pain or discomfort, and 18.5% demonstrated at least moderate anxiety or depression (see Additional file 1: Table S2). At the 12-month follow-up visit, 31.6% of patients experienced at least moderate difficulty with mobility; 8.8% had at least moderate difficulty with self-care; 29% suffered at least moderate difficulty when performing usual activities; 38.6% had at least moderate pain or discomfort, and 15.7% demonstrated at least moderate anxiety or depression (see Additional file 1: Table S3).

As for tumour severity during the baseline period, 37.8% of patients experienced at least moderate difficulty with mobility, 33.3% suffered at least moderate difficulty when performing usual activities, and 40% had at least moderate pain or discomfort in the moderately diffused category. In the severe diffuse category, 36.1% of patients experienced at least moderate difficulty with mobility, 41.7% suffered at least moderate difficulty when performing usual activities, and 48.6% had at least moderate pain or discomfort (see Additional file 1: Table S4). At the 12-month follow-up visit, 32.3% of patients experienced at least moderate difficulty with mobility, 26.5% suffered at least moderate difficulty when performing usual activities, and 32.4% had at least moderate pain or discomfort in the moderately diffused category. In the severe diffuse category, 32.3% of patients experienced at least moderate difficulty with mobility, 30.8% suffered at least moderate difficulty when performing usual activities, and 40% had at least moderate pain or discomfort (see Additional file 1: Table S5).

**Fig. 1:**
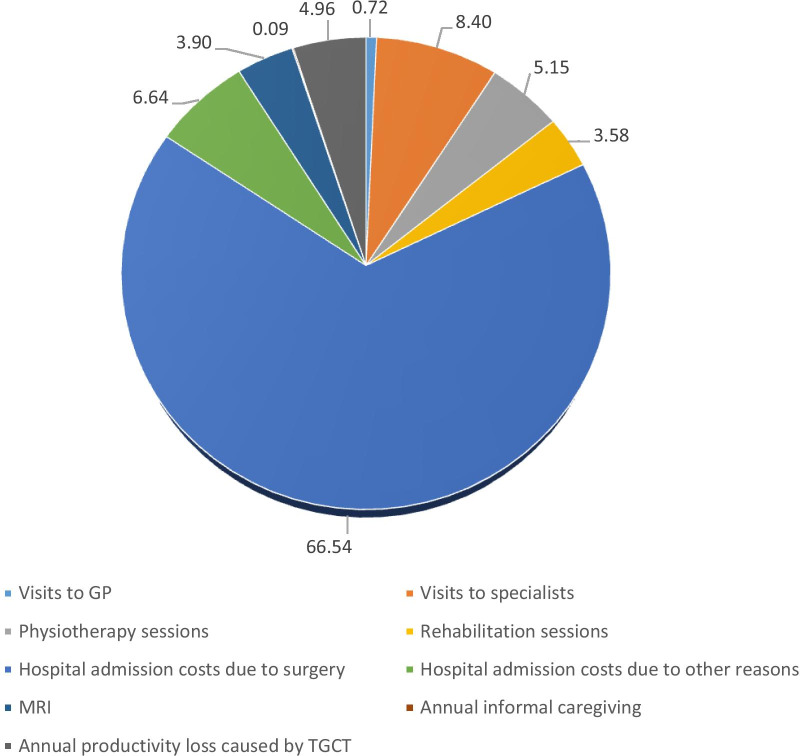
Share of each cost category out of the total cost at baseline. Units: percentage; Note: the percentage represents the weight of each cost category out of the total cost. GP = general practitioner; MRI: Magnetic Resonance Imaging; TGCT: Tenosynovial Giant-Cell Tumour

The use of both healthcare and non-healthcare resources can be translated into an average cost associated with TGCT of €4866 for the baseline period, where healthcare costs represent €4620. Within healthcare costs, those associated with hospitalisations for surgery are the highest (€3238 per patient), followed by the cost of medical visits (€868) (Table 2). Figure 1 represents the weight of each cost category out of the total cost at baseline.

**Table 2:** Average annual costs per patient by country (€2019) during the baseline period

	AUT (n = 9)	GER (n = 12)	SP (n = 13)	FRA (n = 4)	ITA (n = 38)	NLD (n = 61)	ALL (n = 137)
Visits to GPs, mean (sd)	18.86 (34)	75.28 (183.31)	9.37 (24.34)	11.51 (23.02)	47.62 (118.31)	28.17 (47.17)	34.81 (89.13)
Visits to specialists, mean (sd)	155.45 (149.1)	436.18 (354.89)	167.98 (376.71)	29.88 (34.50)	639.22 (860.58)	373.37 (516.46)	408.78 (610.48)
Physiotherapy sessions, mean (sd)	527.24 (907.56)	775.25 (1575.33)	0 (0)	58.30 (116.60)	120.23 (353.33)	253.55 (705.55)	250.49 (737.74)
Rehabilitation sessions (days), mean (sd)	225.74 (677.23)	111.35 (385.74)	0 (0)	0 (0)	130.84 (360.55)	254.46 (1172.26)	174.18 (829.68)
Total medical visit costs, mean (sd)	927.29 (1349.13)	1398.06 (1640.83)	177.36 (395.40)	99.69 (162.01)	937.91 (1159.51)	909.55 (1418.44)	868.25 (1297.20)
Hospital admission costs due to surgery, mean (sd)	9407.31 (5607.22)	7086.28 (4099.31)	–	1249.45 (1448.49)	1712.24 (6859.89)	3455.21 (3591.10)	3237.51 (4624.56)
Hospital admission costs for other reasons, mean (sd)	–	3078.84 (1694.47)	42.83 (100.04)	–	43.69 (679.05)	14.86 (82.33)	323.09 (1362.44)
MRI, mean (sd)	337.06 (157.41)	98.41 (81.56)	182.44 (268.54)	108.68 (125.49)	118.97 (122.15)	237.18 (151.34)	189.85 (166.28)
Direct healthcare costs, mean (sd)	10,669.91 (10,964.53)	11,659.91 (11,637.34)	399.33 (419.42)	1457.82 (1382.75)	2810.02 (5637.92)	4614.32 (8042.43)	4619.61 (8068.00)
Annual informal caregiving, mean (sd)	0 (0)	1.09 (3.76)	0 (0)	0 (0)	0 (0)	9.61 (45.87)	4.38 (30.85)
Annual productivity loss caused by TGCT, mean (sd)	196.14 (537.21)	841.71 (1425.98)	0 (0)	0 (0)	307.95 (648.87)	156.18 (325.11)	241.57 (621.56)
Total costs caused by TGCT, mean (sd)	10,866.04 (11,039.89)	12,502.70 (12,545.13)	399.33 (419.42)	1457.82 (1382.75)	3117.97 (5917.02)	4780.11 (8236.23)	4865.56 (8376.76)

AUT = Austria; GER = Germany; SP = Spain; FRA = France; ITA = Italy; NLD = The Netherlands; MRI: Magnetic Resonance Imaging; TGCT: Tenosynovial Giant-Cell Tumour. Note: ‘Total medical visit costs’ includes the economic valuation of visits to GPs, visits to specialists, physiotherapy sessions and rehabilitation sessions

**Fig. 2:**
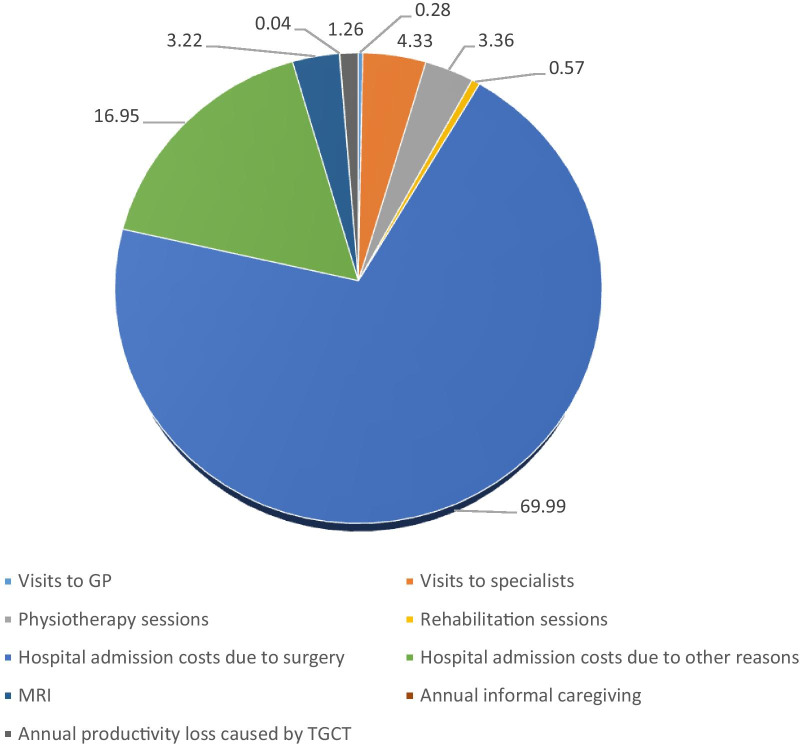
Share of each cost category out of the total cost, at the 12-months visit. Units: percentage; Note: the percentage represents the weight of each cost category out of the total cost. GP = general practitioner; MRI: Magnetic Resonance Imaging; TGCT: Tenosynovial Giant-Cell Tumour

At the 12-month follow-up visit, the average cost was generally higher than in the baseline period. The average cost associated with TGCT was €5160, where €5094 was for healthcare. Within healthcare costs, those associated with hospitalisations for surgery were the highest (€3612) per patient, followed by hospital admissions for other reasons (€874) and medical visits (€441) (Table 3). Figure 2 represents the weight of each cost category out of the total cost at the 12-months follow-up visit.

**Table 3:** Average annual costs per patient by country (€2019) at the 12-month visit

	AUT (n = 9)	GER (n = 12)	SP (n = 13)	FRA (n = 4)	ITA (n = 38)	NLD (n = 61)	ALL (n = 137)
Visits to GPs, mean (sd)	31.43 (94.30)	18.17 (38.63)	0.00 (0.00)	0.00 (0.00)	22.37 (52.79)	10.71* (66.57)	14.63* (58.54)
Visits to specialists, mean (sd)	48.36* (71.42)	227.30 (434.95)	69.99 (153.85)	0.00 (0.00)	152.19* (249.74)	340.78 (340.17)	223.68* (314.72)
Physiotherapy sessions, mean (sd)	271.99 (364.05)	294.60 (716.04)	0.00 (0.00)	17.49 (34.98)	77.24 (233.56)	242.28 (478.41)	173.48 (418.74)
Rehabilitation sessions (days), mean (sd)	0.00 (0.00)	84.84 (293.89)	0.00 (0.00)	0.00 (0.00)	78.80 (238.28)	0.00* (0.00)	29.29* (154.72)
Total medical visit costs, mean (sd)	351.78 (456.74)	624.91 (1,118.68)	69.99 (153.85)	17.49 (34.98)	330.61* (533.57)	593.77 (595.08)	441.08* (620.57)
Hospital admission costs due to surgery, mean (sd)	3200* (5873.22)	1840.72* (4873.27)	0 (0)	0 (0)	1224.78* (4299.81)	6494.20* (2346.02)	3612.02 (3201.29)
Hospital admission costs for other reasons, mean (sd)	890.31 (1568.10)	9185.94* (969.55)	0 (0)	0 (0)	-	48.87* (127.53)	874.48* (1671.24)
MRI, mean (sd)	433.36 (375.30)	131.21 (167.84)	266.64 (338.17)	0.00 (0.00)	99.68 (74.38)	164.20* (185.79)	166.02 (212.68)
Direct healthcare costs, mean (sd)	4875.46 (7902.45)	11,782.78 (28,794.26)	336.64 (312.40)	17.49 (34.98)	1655.20 (4320.65)	7298.48* (7053.83)	5093.60 (10,494.22)
Annual informal caregiving, mean (sd)	0 (0)	0.00 (0.00)	0.00 (0.00)	0.00 (0.00)	6.92* (23.94)	0.00 (0.00)	1.92 (12.87)
Annual productivity loss caused by TGCT, mean (sd)	405.80 (1217.40)	28.06* (57.14)	0.00 (0.00)	0.00 (0.00)	81.69* (255.77)	29.53* (128.92)	64.92* (348.64)
Total costs caused by TGCT, mean (sd)	5281.26 (7819.10)	11,810.84 (28,810.61)	336.64 (312.40)	17.49 (34.98)	1743.81 (4362.88)	7328.01* (7063.49)	5160.44 (10,497.92)

AUT = Austria; GER = Germany; SP = Spain; FRA = France; ITA = Italy; NLD = The Netherlands; LOS: Length Of Stay; MRI: Magnetic Resonance Imaging; TGCT: Tenosynovial Giant-Cell Tumour. Note: ‘Total medical visit cost’ includes the economic valuation of visits to GPs, visits to specialists, physiotherapy sessions and rehabilitation sessions.

*Mean cost was statistically different (p-value < 0.05) from baseline moment according to the two-sample t-test assuming unequal variances

By tumour severity (Additional file 1 S6) during the baseline period, the average cost per individual with severe diffuse-type tumours was €3909. It was €6304 for individuals with moderate diffuse tumours, and €5203 for those with not-assessable tumours. The cost associated with healthcare use was €3771 for individuals with severe diffuse tumours, €5820 for those with moderate diffuse tumours, and €5107 for not–assessable ones. Within healthcare costs, those related to hospitalisation for surgery were the most significant, at €2589, €3915, and €3513 for severe diffuse, moderate diffuse, and not assessable tumours, respectively. Similarly, productivity losses were €135, €484, and €74 for severe diffuse, moderate diffuse, and not assessable tumours, respectively. By tumour severity (Additional file 1 S7) at the 12-month follow-up visit, the average cost per individual with severe diffuse tumours was €5003. It was €5799 for those with moderate diffuse tumours, and €4210 for those with not-assessable tumours. The cost associated with healthcare use was €4962 for those with severe diffuse tumours, €5664 for those with moderate diffuse tumours, and €4206 for not-assessable tumours. Within healthcare costs, those related to hospitalisation due to surgery were the most significant, at €4119, €3129, and €3602 for severe diffuse, moderate diffuse, and not assessable tumours, respectively. Similarly, productivity losses were €40, €131, and €4 for severe diffuse, moderate diffuse, and not assessable tumours, respectively.

## Discussion

Among rare diseases, TGCT is a significant health problem with increasingly important social consequences, especially in high-income countries. The incidence and prevalence of TGCT and its health and social consequences in terms of mortality, morbidity, economic costs, and quality of life justify the attention received from health authorities and the society in general. For this reason, this analysis has focused on (i) determining the use of healthcare resources (i.e., visits to GPs, visits to specialists, physiotherapy sessions, rehabilitation sessions/days, hospitalisations due to surgery and other reasons, MRIs and biopsies), and non-healthcare resources (i.e., working days lost and family caregiving) due to TGCT, (ii) calculating the total cost associated with TGCT, and (iii) assessing the HRQOL at two different time points (baseline and at 12-month follow-up visit).

Regarding healthcare resources, people suffering from TGCT visited specialists and physiotherapists more frequently, especially in countries such as Austria, Germany, and the Netherlands. Similarly, the importance of non-healthcare resource utilisation due to TGCT needs to be highlighted.

The large impact on healthcare costs which our estimates found is consistent with the burden imposed by the disease on TGCT patients in the United States—where one study detailing the annual mean cost-of-illness for TGCT patients found a substantial increase between the baseline and the follow-up period. Mean total healthcare costs increased from $8943 in the baseline to $14,880 in the follow-up period, with more than half the costs covering outpatient care [12]. Another US study detailing indirect costs (productivity loss) per TGCT patient per year showed differences based on whether the TGCT had undergone surgery or not—ranging from $5119 to a maximum of $4403 per year [26]. Although the results are not comparable, US figures show that the economic burden of TGCT is higher in terms of healthcare costs than non-healthcare costs, as our findings also indicate.

By country, the average annual cost per patient during the baseline period was estimated at between €399 (Spain) and €12,503 (Germany). At the 12-month follow-up visit, the average annual cost per patient was estimated at between €17 (France) and €11,811 (Germany). The healthcare costs of TGCT are very similar to those of patients with other rare diseases such as Cystic fibrosis, Prader-Willi syndrome, haemophilia, Duchenne muscular dystrophy, epidermolysis bullosa, Fragile X syndrome, scleroderma, mucopolysaccharidosis, juvenile idiopathic arthritis, and histiocytosis [27]. However, our results suggest that the largest proportion of total and healthcare costs corresponded to hospitalisation due to surgery—due to longer lengths of stay rather than possible re-surgery. Only eight patients from our sample (less than 6%) underwent surgery in both periods. Nevertheless, moderate diffuse tumour patients required more surgical treatments and longer-length hospital stays, thus incurring higher costs than severe diffuse tumour patients.

Another important finding of the study was the burden imposed on the quality of life of TGCT patients. It should be remembered that HRQOL provides information on the overall societal impact of a specific health problem. Its measurement, together with other information sources such as incidence, prevalence, mortality, and costs can be a valuable indicator for setting priorities and allocating healthcare and social resources. Knowledge of HRQOL is also necessary to assess the effectiveness of healthcare interventions in disease management [28].

Our study reviewed HRQOL instruments used for TGCT patients. Four articles describing studies of HRQOL in patients with TGCT were identified [4, 13–15]. The Short Form 36 (SF-36) and Short Form 12 (SF-12) health survey questionnaires and the EQ-5D-5L instrument were the most frequently used, in combination with other specific instruments—i.e., the Western Ontario and McMaster Universities Osteoarthritis Index (WOMAC), the Knee Society Score (KSS), the Harris Hip Score (HHS), the Numerical Rating Scale (NRS) for symptom intensity (pain, stiffness, swelling, immobility, limited motion), and the Patient-Reported Outcomes Measurement Information System (PROMIS).

Our results using the EQ-5D-5L instrument show that HRQOL in TGCT patients is mainly affected in three dimensions: pain or discomfort, mobility, and the performance of usual activities—both at the baseline period and in the 12-month follow-up visit. These three dimensions are worse in TGCT patients than in the general population at the same age: pain or discomfort (22.4% moderate or severe difficulty), mobility (4.2% moderate or severe difficulty), and performance of usual activities (4.1% moderate or severe difficulty) [29].

In terms of HRQOL, TGCT patients reported a 0.75 index score and 68.34 points on the VAS in the baseline period. In the 12-month follow-up visit, TGCT patients reported a 0.76 index score and 71.27 points on the VAS—a slightly better score than in the baseline period. Patients with TGCT reported a relatively low HRQOL compared to the general population at the same age (0.95 index score) [30]. Their HRQOL is also similar to that of patients with chronic illnesses such as cardiovascular diseases (0.75 index score); osteoarthritis, arthritis, or rheumatism (0.77 index score); chronic obstructive pulmonary disease (0.78 index score); diabetes (0.79 index score); stomach or duodenum ulcer (0.83 index score); cirrhosis or liver dysfunction (0.77 index score); malignant tumours (0.79 index score) [29], and other rare diseases such as cystic fibrosis, Prader-Willi syndrome, haemophilia, epidermolysis bullosa, Fragile X syndrome, scleroderma, juvenile idiopathic arthritis, and histiocytosis [27].

Several limitations were found during the analysis due to available data. Direct healthcare costs could be underestimated since we did not assess medication costs. Besides, we only estimated the productivity loss caused by absenteeism (working days missed due to TGCT), since it was not possible to estimate the cost associated with permanent absence—i.e., losses caused by people leaving their job indefinitely due to TGCT. There were several reasons for this. First, there was no information available regarding at what point in the baseline period patients had changed their reported employment situation. Moreover, the variable which contained information on changes in employment status due to TGCT was relatively scarce, and there were often missing values. Regarding the estimates of the amount of care received, several issues must be considered. First, there were some inconsistencies between the information reported in the variables that identified whether the patients required caregiving from their families and the amount of caregiving time they actually received. Secondly, it was found that many people stated that they required informal caregiving. However, few (none in some countries) eventually received it (either no time or zero hours of informal care were reported).

Despite these limitations, we believe that this study represents the most complete and realistic assessment of the economic burden of TGCT patients so far performed in the European context. The main strength of the study lies on its bottom-up approach to costing. In addition, the costs were estimated for a period of one year (baseline and 12 months follow-up). Therefore, they provide an accurate picture of the medium-term burden of TGCT.

Our findings highlight the potential economic burden of TGCT throughout Europe. The information might help decision-makers understand the impact of this disease on society, beyond its consequences for patients and the healthcare system. This information does not replace, but can be complementary to, epidemiological data on disabilities, morbidity, and mortality caused by a disease. However, it is necessary to have a clear understanding of the current patterns of resource utilisation, costs, and HRQOL of TGCT patients to adequately inform healthcare services planning. Although this kind of studies are frequent and growing in number in high-prevalence diseases, this is not the case in rare diseases—due to inherent difficulties in obtaining information about the people affected. Even though efforts have been made in recent years to find more information on the economic burden imposed by rare diseases [27], there is still a serious lack of information on many of them. First, owing to their low prevalence, the correct diagnosis of rare diseases is complex and subject to significant delays. Moreover, most rare diseases have no cure. For many, there is no effective treatment available or, if treatments do exist, there is no guarantee of associated improvements in life expectancy or quality of life.

## Conclusion

We conclude that TGCT places a considerable burden on its sufferers and society, including very high healthcare costs and a deterioration in HRQOL—mostly due to pain or discomfort, impaired mobility, and difficulty in performing usual activities. The information presented here might help decision-makers understand the impact of this disease on society, beyond its consequences for patients and the healthcare system.

## Supplementary Information

**Additional file 1**. Unit costs, health-related quality of life by country and average annual costs per patient by tumour severity.

## Data Availability

The datasets used and/or analysed during the current study are available from the corresponding author on reasonable request.

## Abbreviations

MRI: Magnetic Resonance Imaging
TGCT: Tenosynovial giant-cell tumour
HRQOL: Health-Related Quality of Life
EUROSTAT: Statistical office of the European Union
TOPP: TGCT Observational Platform Project
AUT: Austria
GER: Germany
SP: Spain
FRA: France
ITA: Italy
NLD: The Netherlands
SD: Standard deviation
PROMIS: Patient-Reported Outcomes Measurement Information System
NRS: Numerical Rating Scale
HHS: Harris Hip Score
KSS: The Knee Society Score
WOMAC: Western Ontario and McMaster Universities Osteoarthritis Index
SF-36: Short Form 36
SF-12: Short Form 12
NRS: Numerical Rating Scale
EQ-5D-5L: EuroQol 5-Dimension Questionnaire
VAS: Visual Analogue Scale
