# Supplementary material for: Economic burden and health-related quality of life in tenosynovial giant-cell tumour patients in Europe: an observational disease registry
Source: Orphanet J Rare Dis. 2021 Jul 2;16:294. doi: 10.1186/s13023-021-01883-5 (PMC8254314; doi:10.1186/s13023-021-01883-5)
Supplement: Supplementary file 1 — Additional file 1. Unit costs, health-related quality of life by country and average annual costs per patient by tumour severity. [file 13023_2021_1883_MOESM1_ESM.docx]

Table S1: Unit Costs by Country (Inflated to 2019 EUR)

| **Resource use items** | Austria | France | Germany | Italy | Netherland | Spain |
| --- | --- | --- | --- | --- | --- | --- |
| Visits to GP (source) | 18.66 (1) | 23.02 (2) | 31.15 (3) | 21.80 (4) | 34.37 (5) | 40.62 (6) |
| Visits to specialist (source) | 31.09 (1) | 29,88 (2) | 73.72 (7) | 128.52 (7) | 82.82 (5) | 90.99 (6) |
| Rehabilitation session (source) | 145.12 (1) |  | 132.44 (8) | 128.90 (8) | 158.39 (5) |  |
| Physiotherapy session (source) | 37.66 (1) | 23.32 (2) | 62.02 (3) | 27.69 (4) | 34.37 (5) |  |
| MRI (source) | 216.68 (1) | 217.36 (2) | 131.21 (9) | 122.19 (4) | 222.58 (5) | 182.44 (6) |
| Hospital bed day (source) |  |  | 772.3 (10) | 452.7 (10) | 657 (10) |  |
| Radiation therapy (source) | 1,405 (10) |  | 5,147 (10) |  |  |  |
| Biopsy (source) |  |  |  | 151.05 (10) | 151.05 (10) | 257 (10) |
| Arthroscopic resection/synovectomy: knee joint (source) | 10,350 (10) | 2,338.20 (10) |  | 1,436.14 (10) | 15,000 (10) |  |
| Open resection/one-stage synovectomy: knee joint (source) | 13,400 (10) | 2,659.58 (10) |  | 4,648.19 (10) | 10,000 (10) |  |
| Open resection/one-stage synovectomy: ankle joint (source) | 10,350 (10) |  |  |  | 10,000 (10) |  |
| Open resection/two-stage synovectomy: knee joint (source) | 15,400 (10) |  |  |  | 15,000 (10) |  |
| Open resection/one-stage synovectomy: elbow joint (source) |  |  |  |  | 10,000 (10) |  |
| Open resection/one-stage synovectomy: foot (source) |  |  | 10,000 (10) |  |  |  |
| (Tumor) Prosthesis: knee joint (source) |  |  | 17,800 (10) |  | 25,000 (10) |  |
| Anterior en posterior synovectomy: knee joint (source) |  |  |  |  | 15,000 (10) |  |
| Resection of TGCT and total hip prosthes (source) |  |  |  |  | 15,000 (10) |  |
| Resection of subdeltoid bursitis: shoulder joint (source) |  |  |  |  | 7,000 (10) |  |
| synovectomy and total knee prosthesis: knee joint (source) |  |  |  |  | 10,000 (10) |  |
| Hourly wage by sex and age (source) | 12.54* - 27.21** (11) |  | 12.65* -22.82** (11) | 10.67* – 23.95** (11) | 11.69* - 22.3** (11) |  |
| Informal care unit cost according to the proxy good method (source) | 34.81 (12) |  | 14.82 (12) | 9.51 (12) | 13.16 (13) |  |

*hourly wage for a woman of 15-29 years old ; **hourly wage for a man of 60-64 years;

(1) Mayer S, Kiss N, Laszewska A, Simo, J (2017): Costing evidence for health care decission-making in Austria: A systematic review. PLOS ONE, 12(8): e0183116

(2) Sécurité Sociale l’Assurance Maladie [website] Avialable at: <http://www.ameli.fr/professionnels-de-sante/medecins/votre-convention/tarifs/tarifs-conventionnels-des-medecins-generalistes/tarifs-des-medecins-generalistes-en-metropole.php>

(3) Einheitlicher Bewertungsmaßstab (EBM). Kassenärztliche Bundesvereinigung. Available at: <https://www.kbv.de/html/index.php>

(4) Gazzetta Ufficiale della Repubblica Italiana, Supplemento ordinario n. 8. Serie generale - n. 23 (28-1-2013) Available at: <http://www.trovanorme.salute.gov.it/norme/renderPdf.spring?seriegu=SG&datagu=28/01/2013&redaz=13A00528&artp=3&art=1&subart=1&subart1=10&vers=1&prog=001>

(5) Kanters TA, Bouwmans CAM, van der Linden N, Tan SS, Hakkaart-van Roijen L (2017): Update of the Dutch manual for costing studies in health care. PLoS ONE 12(11): e0187477.

(6) Official Autonomic Gazzettes. Avilable at: <https://www.boe.es/legislacion/otros_diarios_oficiales.php?lang=en>

(7) López-Bastida J, Oliva-Moreno J, Linertová R, Serrano-Aguilar P. Social/economic costs and health-related quality of life in patients with rare diseases in Europe. Eur J Health Econ. 2016 Apr;17 Suppl 1:1-5.

(8) Verleger K, Penrod JR, Manley Daumont M, Solem C, Luo L, Macahilig C, Hertel N. Costs and Cost Drivers Associated with Non-Small-Cell Lung Cancer Patients Who Received Two or More Lines of Therapy in Europe. Clinicoecon Outcomes Res. 2020 Jan 15;12:23-33.

(9) Kassenärztliche Bundesvereinigung. Available at: <https://www.kbv.de/html/index.php>

(10) Local (national) hospital accounting department

(11) Eurostat. Structure of earnings survey. Available at: <https://ec.europa.eu/eurostat/web/products-datasets/-/earn_ses_hourly>

(12) European Healthcare and social costs database (EU HCS€D). Available at: <https://www.easp.es/Impact-Hta/Default>

(13) IJzerman, M.J. Guideline for economic evaluations in healthcare (2016). Available at: <https://english.zorginstituutnederland.nl/publications/reports/2016/06/16/guideline-for-economic-evaluations-in-healthcare>

Table S2: Health-related quality of life (HRQOL) of patients by country at a pre-baseline moment

|  |  | AUT (n=9) | GER (n=12) | SP (n=12) | FRA (n=4) | ITA (n=38) | NLD (n=60) | ALL (n=135) |
| --- | --- | --- | --- | --- | --- | --- | --- | --- |
| Mobility | I have no problems in walking about | 44.40% | 16.70% | 41.70% | 75.00% | 42.10% | 16.70% | 29.60% |
|  | I have slight problems in walking about | 33.30% | 50.00% | 33.30% | 0.00% | 23.70% | 36.70% | 32.60% |
|  | I have moderate problems in walking about | 11.10% | 16.70% | 16.70% | 25.00% | 18.40% | 43.30% | 28.90% |
|  | I have severe problems in walking about | 11.10% | 16.70% | 8.30% | 0.00% | 15.80% | 1.70% | 8.10% |
|  | I am unable to move | 0.00% | 0.00% | 0.00% | 0.00% | 0.00% | 1.70% | 0.70% |
| Self-care | I have no problems washing or dressing myself | 88.90% | 66.70% | 66.70% | 75.00% | 71.10% | 78.30% | 74.80% |
|  | I have slight problems washing or dressing myself | 0.00% | 8.30% | 16.70% | 0.00% | 18.40% | 15.00% | 14.10% |
|  | I have moderate problems washing or dressing myself | 11.10% | 16.70% | 16.70% | 25.00% | 5.30% | 6.70% | 8.90% |
|  | I have severe problems washing or dressing myself | 0.00% | 8.30% | 0.00% | 0.00% | 5.30% | 0.00% | 2.20% |
|  | I am unable to wash or dress myself | 0.00% | 0.00% | 0.00% | 0.00% | 0.00% | 0.00% | 0.00% |
| Usual activities | I have no problems doing my usual activities | 55.60% | 33.30% | 50.00% | 75.00% | 34.20% | 25.00% | 34.10% |
|  | I have slight problems doing my usual activities | 11.10% | 16.70% | 33.30% | 0.00% | 31.60% | 33.30% | 28.90% |
|  | I have moderate problems doing my usual activities | 11.10% | 41.70% | 8.30% | 25.00% | 26.30% | 31.70% | 27.40% |
|  | I have severe problems doing my usual activities | 11.10% | 8.30% | 0.00% | 0.00% | 7.90% | 8.30% | 7.40% |
|  | I am unable to do my usual activities | 11.10% | 0.00% | 8.30% | 0.00% | 0.00% | 1.70% | 2.20% |
| Pain/ Discomfort | I have no pain or discomfort | 33.30% | 8.30% | 0.00% | 25.00% | 18.40% | 10.00% | 13.30% |
|  | I have slight pain or discomfort | 22.20% | 25.00% | 58.30% | 25.00% | 39.50% | 38.30% | 37.80% |
|  | I have moderate pain or discomfort | 44.40% | 33.30% | 33.30% | 50.00% | 18.40% | 40.00% | 33.30% |
|  | I have severe pain or discomfort | 0.00% | 33.30% | 8.30% | 0.00% | 23.70% | 10.00% | 14.80% |
|  | I have extreme pain or discomfort | 0.00% | 0.00% | 0.00% | 0.00% | 0.00% | 1.70% | 0.70% |
| Anxiety/ Depression | I am not anxious or depressed | 55.60% | 33.30% | 50.00% | 50.00% | 65.80% | 68.30% | 61.50% |
|  | I am slightly anxious or depressed | 22.20% | 16.70% | 25.00% | 0.00% | 18.40% | 21.70% | 20.00% |
|  | I am moderately anxious or depressed | 11.10% | 25.00% | 25.00% | 50.00% | 15.80% | 6.70% | 14.10% |
|  | I am severely anxious or depressed | 11.10% | 25.00% | 0.00% | 0.00% | 0.00% | 3.30% | 4.40% |
|  | I am extremely anxious or depressed | 0.00% | 0.00% | 0.00% | 0.00% | 0.00% | 0.00% | 0.00% |

AUT=Austria; GER=Germany; SP=Spain; FRA=France; ITA=Italy; NLD=The Netherlands

Table S3. Health-related quality of life (HRQOL) of patients by country at the 12-months visit

|  |  | AUT (n=9) | GER (n=12) | SP (n=12) | FRA (n=4) | ITA (n=38) | NLD (n=60) | ALL (n=135) |
| --- | --- | --- | --- | --- | --- | --- | --- | --- |
| Mobility | I have no problems in walking about | 50.00% | 27.30% | 58.30% | 75.00% | 30.00% | 34.70% | 37.70% |
|  | I have slight problems in walking about | 37.50% | 18.20% | 41.70% | 0.00% | 40.00% | 26.50% | 30.70% |
|  | I have moderate problems in walking about | 0.00% | 36.40% | 0.00% | 25.00% | 23.30% | 20.40% | 19.30% |
|  | I have severe problems in walking about | 12.50% | 18.20% | 0.00% | 0.00% | 6.70% | 18.40% | 12.30% |
|  | I am unable to move | 0.00% | 0.00% | 0.00% | 0.00% | 0.00% | 0.00% | 0.00% |
| Self-care | I have no problems washing or dressing myself | 87.50% | 54.50% | 75.00% | 75.00% | 63.30% | 85.70% | 75.40% |
|  | I have slight problems washing or dressing myself | 0.00% | 18.20% | 16.70% | 25.00% | 26.70% | 10.20% | 15.80% |
|  | I have moderate problems washing or dressing myself | 0.00% | 18.20% | 8.30% | 0.00% | 6.70% | 4.10% | 6.10% |
|  | I have severe problems washing or dressing myself | 12.50% | 0.00% | 0.00% | 0.00% | 0.00% | 0.00% | 0.90% |
|  | I am unable to wash or dress myself | 0.00% | 9.10% | 0.00% | 0.00% | 3.30% | 0.00% | 1.80% |
| Usual activities | I have no problems doing my usual activities | 62.50% | 27.30% | 58.30% | 75.00% | 40.00% | 34.70% | 41.20% |
|  | I have slight problems doing my usual activities | 12.50% | 45.50% | 41.70% | 0.00% | 26.70% | 30.60% | 29.80% |
|  | I have moderate problems doing my usual activities | 12.50% | 9.10% | 0.00% | 25.00% | 26.70% | 24.50% | 20.20% |
|  | I have severe problems doing my usual activities | 12.50% | 18.20% | 0.00% | 0.00% | 3.30% | 8.20% | 7.00% |
|  | I am unable to do my usual activities | 0.00% | 0.00% | 0.00% | 0.00% | 3.30% | 2.00% | 1.80% |
| Pain/Discomfort | I have no pain or discomfort | 37.50% | 18.20% | 8.30% | 25.00% | 13.30% | 10.20% | 14.00% |
|  | I have slight pain or discomfort | 25.00% | 18.20% | 75.00% | 50.00% | 56.70% | 44.90% | 47.40% |
|  | I have moderate pain or discomfort | 25.00% | 36.40% | 16.70% | 0.00% | 23.30% | 32.70% | 27.20% |
|  | I have severe pain or discomfort | 12.50% | 18.20% | 0.00% | 25.00% | 3.30% | 12.20% | 9.60% |
|  | I have extreme pain or discomfort | 0.00% | 9.10% | 0.00% | 0.00% | 3.30% | 0.00% | 1.80% |
| Anxiety/Depression | I am not anxious or depressed | 37.50% | 36.40% | 66.70% | 25.00% | 60.00% | 69.40% | 59.60% |
|  | I am slightly anxious or depressed | 25.00% | 18.20% | 33.30% | 50.00% | 23.30% | 22.40% | 24.60% |
|  | I am moderately anxious or depressed | 25.00% | 27.30% | 0.00% | 25.00% | 6.70% | 6.10% | 9.60% |
|  | I am severely anxious or depressed | 12.50% | 18.20% | 0.00% | 0.00% | 10.00% | 2.00% | 6.10% |
|  | I am extremely anxious or depressed | 0.00% | 0.00% | 0.00% | 0.00% | 0.00% | 0.00% | 0.00% |

AUT=Austria; GER=Germany; SP=Spain; FRA=France; ITA=Italy; NLD=The Netherlands

Table S4: Health-related quality of life (HRQOL) of patients by tumour severity in the pre-baseline moment

|  |  | Not assessable (n=18) | Moderately diffused (n=45) | Severely diffused (n=72) | Total (n=135) |
| --- | --- | --- | --- | --- | --- |
| Mobility | I have no problems in walking about | 33,30% | 24,40% | 31,90% | 29,60% |
|  | I have slight problems in walking about | 22,20% | 37,80% | 31,90% | 32,60% |
|  | I have moderate problems in walking about | 27,80% | 28,90% | 29,20% | 28,90% |
|  | I have severe problems in walking about | 11,10% | 8,90% | 6,90% | 8,10% |
|  | I am unable to move | 5,60% | 0,00% | 0,00% | 0,70% |
| Self-care | I have no problems washing or dressing myself | 77,80% | 77,80% | 72,20% | 74,80% |
|  | I have slight problems washing or dressing myself | 0,00% | 15,60% | 16,70% | 14,10% |
|  | I have moderate problems washing or dressing myself | 16,70% | 6,70% | 8,30% | 8,90% |
|  | I have severe problems washing or dressing myself | 5,60% | 0,00% | 2,80% | 2,20% |
|  | I am unable to wash or dress myself | 0,00% | 0,00% | 0,00% | 0,00% |
| Usual activities | I have no problems doing my usual activities | 44,40% | 33,30% | 31,90% | 34,10% |
|  | I have slight problems doing my usual activities | 27,80% | 33,30% | 26,40% | 28,90% |
|  | I have moderate problems doing my usual activities | 22,20% | 24,40% | 30,60% | 27,40% |
|  | I have severe problems doing my usual activities | 0,00% | 6,70% | 9,70% | 7,40% |
|  | I am unable to do my usual activities | 5,60% | 2,20% | 1,40% | 2,20% |
| Pain/ Discomfort | I have no pain or discomfort | 5,60% | 13,30% | 15,30% | 13,30% |
|  | I have slight pain or discomfort | 22,20% | 46,70% | 36,10% | 37,80% |
|  | I have moderate pain or discomfort | 55,60% | 24,40% | 33,30% | 33,30% |
|  | I have severe pain or discomfort | 16,70% | 15,60% | 13,90% | 14,80% |
|  | I have extreme pain or discomfort | 0,00% | 0,00% | 1,40% | 0,70% |
| Anxiety/ Depression | I am not anxious or depressed | 50,00% | 64,40% | 62,50% | 61,50% |
|  | I am slightly anxious or depressed | 22,20% | 17,80% | 20,80% | 20,00% |
|  | I am moderately anxious or depressed | 27,80% | 11,10% | 12,50% | 14,10% |
|  | I am severely anxious or depressed | 0,00% | 6,70% | 4,20% | 4,40% |
|  | I am extremely anxious or depressed | 0,00% | 0,00% | 0,00% | 0,00% |

Table S5: Health-related quality of life (HRQOL) of patients by tumour severity at the 12-months visit

|  |  | Not assessable (n=15) | Moderately diffused (n=34) | Severely diffused (n=65) | Total (n=114) |
| --- | --- | --- | --- | --- | --- |
| Mobility | I have no problems in walking about | 40,00% | 29,40% | 41,50% | 37,70% |
|  | I have slight problems in walking about | 33,30% | 38,20% | 26,20% | 30,70% |
|  | I have moderate problems in walking about | 6,70% | 29,40% | 16,90% | 19,30% |
|  | I have severe problems in walking about | 20,00% | 2,90% | 15,40% | 12,30% |
|  | I am unable to move | 0,00% | 0,00% | 0,00% | 0,00% |
| Self-care | I have no problems washing or dressing myself | 66,70% | 70,60% | 80,00% | 75,40% |
|  | I have slight problems washing or dressing myself | 13,30% | 23,50% | 12,30% | 15,80% |
|  | I have moderate problems washing or dressing myself | 13,30% | 5,90% | 4,60% | 6,10% |
|  | I have severe problems washing or dressing myself | 0,00% | 0,00% | 1,50% | 0,90% |
|  | I am unable to wash or dress myself | 6,70% | 0,00% | 1,50% | 1,80% |
| Usual activities | I have no problems doing my usual activities | 53,30% | 32,40% | 43,10% | 41,20% |
|  | I have slight problems doing my usual activities | 20,00% | 41,20% | 26,20% | 29,80% |
|  | I have moderate problems doing my usual activities | 13,30% | 26,50% | 18,50% | 20,20% |
|  | I have severe problems doing my usual activities | 6,70% | 0,00% | 10,80% | 7,00% |
|  | I am unable to do my usual activities | 6,70% | 0,00% | 1,50% | 1,80% |
| Pain/ Discomfort | I have no pain or discomfort | 6,70% | 11,80% | 16,90% | 14,00% |
|  | I have slight pain or discomfort | 46,70% | 55,90% | 43,10% | 47,40% |
|  | I have moderate pain or discomfort | 33,30% | 26,50% | 26,20% | 27,20% |
|  | I have severe pain or discomfort | 6,70% | 5,90% | 12,30% | 9,60% |
|  | I have extreme pain or discomfort | 6,70% | 0,00% | 1,50% | 1,80% |
| Anxiety/ Depression | I am not anxious or depressed | 53,30% | 58,80% | 61,50% | 59,60% |
|  | I am slightly anxious or depressed | 13,30% | 32,40% | 23,10% | 24,60% |
|  | I am moderately anxious or depressed | 26,70% | 5,90% | 7,70% | 9,60% |
|  | I am severely anxious or depressed | 6,70% | 2,90% | 7,70% | 6,10% |
|  | I am extremely anxious or depressed | 0,00% | 0,00% | 0,00% | 0,00% |

Table S6: Average annual costs per patient by tumour severity (€2019) in the pre-baseline period.

|  | Not assessable (n=18) | Moderately diffused (n=45) | Severely diffused (n=74) | Whole sample (n=137) |
| --- | --- | --- | --- | --- |
| Visits to GP, mean (sd) | 27.59 (33.55) | 50.83 (136.67) | 26.82 (55.17) | 34.81 (89.13) |
| Visits to specialists, mean (sd) | 190.64 (312.79) | 483.97 (796.75) | 416.11 (523.09) | 408.78 (610.48) |
| Physiotherapy sessions, mean (sd) | 373.81 (706.84) | 250.10 (877.36) | 220.72 (654.97) | 250.49 (737.74) |
| Rehabilitation sessions (days), mean (sd) | 799.23 (2014.59) | 165.38 (579.04) | 27.49 (130.42) | 174.18 (829.68) |
| **Total medical visit costs, mean (sd)** | **1,391.27 (2,163.75)** | **950.28 (1,371.25)** | **691.14 (898.85)** | **868.25 (1,297.20)** |
| Hospital admission costs due to surgery, mean (sd) | 3,512.57 (8,673.24) | 3,914.66 (8,136.72) | 2,589.34 (6,991.44) | 3,246.98 (4,624.56) |
| Hospital admission costs due to other reasons, mean (sd) | 64.81 (82.22) | 799,87 (1,521.68) | 266.80 (800.31) | 313.39 (1,362.44) |
| MRI, mean (sd) | 141.02 (165.88) | 155.09 (123.53) | 222.87 (182.94) | 189.85 (166.28) |
| **Direct healthcare costs, mean (sd)** | **5,107.28 (8,525.18)** | **5,819.90 (8,439.32)** | **3,771.09 (7,729.06)** | **4,619.61 (8,068.00)** |
| Annual informal caregiving, mean (sd) | 21.22 (77.47) | 0.29 (1.94) | 2.76 (17.04) | 4.38 (30.85) |
| Annual productivity loss caused by TGCT, mean (sd) | 74.42 (262.63) | 484.20 (946.57) | 134.68 (329.95) | 241.57 (621.56) |
| **Total costs caused by TGCT, mean (sd)** | **5,202.92 (8,657.40)** | **6,304.39 (8,955.38)** | **3,908.53 (7,918.45)** | **4,865.56 (8,376.76)** |

MRI: Magnetic Resonance Imaging; TGCT: Tenosynovial Giant-Cell Tumour.

Table S7: Average annual costs per patient by tumour severity (€2019) at the 12-months visit

|  | Not assessable (n=18) | Moderately diffused (n=45) | Severely diffused (n=74) | Whole sample (n=137) |
| --- | --- | --- | --- | --- |
| Visits to GP, mean (sd) | 38 (121.26) | 18.54 (55.29) | 6.57 (30.13) | 14.63 (58.54) |
| Visits to specialists, mean (sd) | 180.24 (228.64) | 151.02 (305.08) | 278.43 (330.91) | 223.68 (314.72) |
| Physiotherapy sessions, mean (sd) | 265.04 (497.98) | 103.00 (366.77) | 194.08 (426.95) | 173.48 (418.74) |
| Rehabilitation sessions (days), mean (sd) | 3.14 (13.32) | 32.67 (158.93) | 33.59 (170.70) | 29.29 (154.72) |
| **Total medical visit costs, mean (sd)** | **486.42 (628.15)** | **305.23 (645.03)** | **512.67 (598.09)** | **441.08 (620.57)** |
| Hospital admission costs due to surgery, mean (sd) | 3,601.89 (2,982.91) | 3,128.78 (3,416.23) | 4,119.08 (3,116.48) | 3,613.02 (3,201.29) |
| Hospital admission costs due to other reasons, mean (sd) | - | 2,090.62 (2,216.45) | 137.84 (1,139.57) | 872.48 (1,671.24) |
| MRI, mean (sd) | 120.04 (223.6) | 140.31 (160.32) | 192.85 (235.54) | 166.02 (212.68) |
| **Direct healthcare costs, mean (sd)** | **4,206.35 (6,619.61)** | **5,664.24 (15,787.46)** | **4,962.41 (6,657.72)** | **5,093.60 (10,494.22)** |
| Annual informal caregiving, mean (sd) | 0 (0) | 3.89 (18.26) | 1.18 (10.19) | 1.92 (12.87) |
| Annual productivity loss caused by TGCT, mean (sd) | 3.59 (15.22) | 131.30 (582.24) | 39.48 (133.30) | 64.92 (348.64) |
| **Total costs caused by TGCT, mean (sd)** | **4,209.94 (6,618.27)** | **5,799.43 (15,774.16)** | **5,003.07 (6,681.30)** | **5,160.44 (1,0497.92)** |

MRI: Magnetic Resonance Imaging; TGCT: Tenosynovial Giant-Cell Tumour.
